# The combined association of STOPPFall medication use and orthostatic blood pressure abnormalities with future falls and fractures in community-dwelling older people

**DOI:** 10.1007/s41999-026-01473-3

**Published:** 2026-04-13

**Authors:** Kate Doyle, Siobhan Scarlett, Silvin P. Knight, Frank Moriarty, Amanda Lavan, Robert Briggs, Rose-Anne Kenny

**Affiliations:** 1 The Irish Longitudinal Study On Ageing (TILDA), Trinity College Dublin, Dublin, Ireland; 2 Mercer’s Institute for Successful Ageing, St James’s Hospital, Dublin, Ireland; 3 Department of Medical Gerontology, Trinity College Dublin, Dublin, Ireland; 4 School of Pharmacy and Biomolecular Sciences, RCSI University of Medicine and Health Sciences, Dublin, Ireland

**Keywords:** Fall-risk-increasing drugs, Deprescribing, Injurious falls, Orthostatic hypotension, Blood pressure, Older people

## Abstract

**Purpose:**

STOPPFall(Screening Tool of Older Persons Prescriptions in older adults with high fall risk) defines fall-risk-increasing drugs(FRIDs) associated with orthostatic hypotension(OH)/falls. This study assesses the association between the cumulative effect of STOPPFall medications and OH, on future falls/fractures, over 4-year follow-up among community-dwelling older people ≥ 65 years using The Irish Longitudinal Study on Ageing(TILDA) data.

**Methods:**

STOPPFall medications were recorded at Wave1. Orthostatic blood pressure(BP) was measured by active stand using a Finometer at Wave1, recording delayed BP recovery(drop in systolicBP(sBP) ≥ 20 mmHg and/or diastolicBP ≥ 10 mmHg from baseline at 30 s post-stand) and “any OH”(same BP drop at any of 30,60,90,120 s post-stand). Falls/fractures were self-reported. Logistic regression models using interaction terms generated odds ratios(ORs) assessing the association between the interaction of STOPPFall medications and delayed BP recovery/any OH at baseline, and falls(including injurious/unexplained falls) and all fractures at follow-up.

**Results:**

1390 participants were included, mean age at baseline 71.0 years, 51% female. One STOPPFall medication was prescribed in 27%(372/1390) participants, 11%(147/1390) were prescribed ≥ 2STOPPFall medications. Over 40% participants fell during follow-up(592/1390,42.6%), 12%(160/1390) sustained fractures. The cumulative effect of ≥ 2STOPPFall medications and delayed BP recovery was independently associated with all falls[OR1.93(95%CI1.04–3.59);*p* = 0.036], injurious falls[OR2.97(95%CI1.55–5.68);*p* = 0.001] and all fractures[OR3.50(95%CI1.66–7.41);*p* = 0.001]. Prescription of ≥ 2STOPPFall medications and any OH was independently associated with all falls[OR1.98(95%CI1.11–3.54);*p* = 0.021], injurious falls[OR2.25(95%CI1.21–4.20);*p* = 0.011] and all fractures[OR2.81(95%CI1.34–5.90);*p* = 0.006].

**Conclusion:**

Prescription of ≥ 2 STOPPFall medications and delayed BP recovery, and prescription of ≥ 2 STOPPFall medications and any OH, was associated with all falls, injurious falls and all fractures. This highlights the importance of multidomain interventions during multifactorial falls assessments.

**Supplementary Information:**

The online version contains supplementary material available at 10.1007/s41999-026-01473-3.

## Key Summary Points

**Aim:**

To examine the cumulative effect of STOPPFall medication use and orthostatic hypotension on future falls and fractures in a large cohort of community-dwelling older people.

**Findings:**

Prescription of ≥2 STOPPFall medications and delayed blood pressure recovery at 30 seconds, and prescription of ≥2 STOPPFall medications and any OH (OH at any of 30, 60, 90 or 120 seconds after standing), was associated with all falls, injurious falls and all fractures.

**Message:**

This study highlights the importance of a comprehensive multifactorial falls assessment with multidomain interventions among older people at risk of falls to reduce future burden of falls and fractures, as part of a comprehensive geriatric assessment.

**Supplementary Information:**

The online version contains supplementary material available at 10.1007/s41999-026-01473-3.

## Introduction

Falls commonly occur among older people and can have deleterious effects on overall health and quality of life, with associated increased risk of fall-related injuries, morbidity and mortality [1, 2]. Falls causing fractures are a significant concern due to subsequent adverse outcomes including functional decline [3], nursing home admission [4] and mortality, with the one-year mortality rate post-hip fracture in Ireland reported as 24% [5].

Modifiable risk factors can contribute to falls among older people, and identifying and optimising risk factors, or combinations of factors, is an essential aspect of multifactorial fall risk assessment and prevention strategies [6]. Two important falls risk factors in older people are orthostatic hypotension (OH) [7] and medication usage, particularly fall-risk-increasing drugs (FRIDs) [6].

OH, defined as a sustained drop in systolic blood pressure (BP) from baseline values ≥ 20 mmHg, drop in diastolic BP ≥ 10 mmHg, or decrease in systolic BP to < 90 mmHg on standing [8], is common among older people. It affects one in five community-dwelling older people aged ≥ 60 years [9], increases with older age, and is associated with an increased risk of falls [10] and incident fractures [11].

STOPPFall (Screening Tool of Older Persons Prescriptions in older adults with high fall risk) defines FRIDs from fourteen different medication classes, developed by Delphi consensus [12]. Prescription of two or more STOPPFall medications is independently associated with an increased likelihood of falls and fractures [13], significantly lower systolic BP at 30, 60, 90 and 120 s post standing and OH [14]. It is not clear, however, whether prescription of STOPPFall medications and OH have a cumulative effect on falls and fracture risk when these two factors are both present, beyond the risk that each factor in isolation may confer. Assessing how these two established falls risk factors may interact with each other is important, especially given the most common cause of non-neurogenic OH in older people is medications.

The aim of this study therefore is to assess the effect of the interactions between STOPPFall medication use and OH on falls and fractures in a large population-based cohort of community-dwelling older people over a 4 year follow-up period.

## Methods

### Study design and participants

This longitudinal study utilises data from The Irish Longitudinal Study on Ageing (TILDA). TILDA is a large, population-based nationally representative sample of community-dwelling older adults in Ireland, aged ≥ 50 years. Waves of data collection are performed every 2 years. This study used data from Waves 1 – 3, collected between 2009 – 2015.

The TILDA study design has been outlined previously [15]. Briefly, there are three components to data collection: a computer-assisted personal interview (CAPI) completed by social interviewers in the participants’ own home; a self-completion questionnaire completed and returned by the participant; and comprehensive centre-based health assessment or modified home-based health assessment carried out by trained research nurses. The health assessment data used in this study was completed at Wave 1 only.

Participants in this study were included if they were aged ≥ 65 years, had a medication list available to assess for medications included in STOPPFall, completed a health assessment at Wave 1 including active stand measuring orthostatic BP, and completed at least 2 years follow-up i.e. to at least Wave 2 of TILDA. Participants were excluded at Wave 1 if a pre-existing diagnosis of dementia was present. This study examines the longitudinal relationship between STOPPFall medications, in the presence or absence of concurrent OH, and subsequent falls and fractures. This study builds on previous work assessing the association between STOPPFall medication use and falls and fractures [13], and the association between STOPPFall medication use and OH [14], following similar methodologies.

### Orthostatic blood pressure measurement

Orthostatic BP was measured by active stand testing using a Finometer® Midi as described previously [16], allowing beat-to-beat finger BP measurement and detailing more sensitive orthostatic BP changes compared to measurement of orthostatic brachial artery BP by sphygmomanometer [17]. The active stand commenced with participants resting supine for ten minutes in a quiet room, then standing promptly assisted by a research nurse if required, and maintaining a standing position during the remaining recording period. Systolic BP (sBP), diastolic BP and heart rate were continuously measured at baseline while supine, and for a further 120 s after standing, recorded at 10-s intervals, with time-point values being 9-s moving averages.

Delayed BP recovery, as previously assessed within TILDA [18], was defined as a drop in sBP ≥ 20 mmHg and/or drop in diastolic BP ≥ 10 mmHg at 30 s after standing. Delayed BP recovery at 30 s has previously been demonstrated to be associated with fractures [18], depression [19], and lower cognitive performance [20] within the TILDA cohort. Given that it is recognised as an important subtype of OH [21, 22], this definition was included in this analysis. Participants were defined as having “any OH” by a drop in sBP ≥ 20 mmHg and/or drop in diastolic BP ≥ 10 mmHg at any of 30, 60, 90 or 120 s after standing.

### STOPPFall medications

Medication lists were examined for all medications within STOPPFall at Wave 1 (baseline) assessment, identified by Anatomical Therapeutic Chemical (ATC) Classification System.

A comprehensive list of ATC codes for the specific medications within thirteen of fourteen medication classes were provided by the creators of STOPPFall, excluding anticholinergic medication, as an international Delphi consensus effort defining medications within this class is pending. The thirteen other STOPPFall medication classes comprise benzodiazepines (identified with ATC codes N03AE, N05BA and N05CD), antipsychotics (ATC code N05A), benzodiazepine-related drugs (N05CF), opioids (N02A, R05DA), antidepressants (N06A, N06CA), antiepileptics (N03), diuretics (C02L, C03, C07B, C07C, C07D, C08GA, C09BA, C09BX01, C09BX03, C10BX13, C09DA, C09DX01, C09DX03, C09DX06, C09DX07, C09XA52, C09XA54), alpha-blockers used as antihypertensives (C02CA), alpha blockers used for prostate hyperplasia (G04CA), centrally-acting antihypertensives (C02A), antihistamines (N07CA02, R06), vasodilators used in cardiac diseases (C01D), and drugs for urinary frequency and incontinence (G04BD, G04CA53). Anticholinergic medications were defined by a comprehensive list of medications with ‘definite’ anticholinergic effects based on the Anticholinergic Cognitive Burden (ACB) scale [23] (i.e., a score of 3), which has been used in previous studies of anticholinergic medication within TILDA [24]. Only anticholinergics not already classified in other STOPPFall classes were included to avoid duplication and included gastrological agents (A03AA07 A03AB05 A03BA01 A03BA03 A03BB01), Parkinsonian agents (N04AA01 N04AA02 N04AA04 N04AB02 N04AC01), antihistamines (N02BE51) and other medications (M03BA03 M03BC01 N05CM05 N05BB01).

### Falls and fractures

At Waves 2 and 3, participants were asked ‘Have you had any falls since the last interview?’, which was used to create the 'Falls' variable. The ‘Unexplained Falls’ variable was defined by a positive answer to the follow-up question ‘Were any of these falls non-accidental, i.e. with no apparent or obvious reason?’ The ‘Injurious Falls’ variable was defined by a positive answer to ‘Did you injure yourself seriously enough to need medical treatment?’.

Fracture data were obtained by self-report. Previous fracture at Wave 1 was self-reported. Participants were asked about hip, wrist, vertebral and other fractures since the last interview at Waves 2 and 3, and positive answer to these questions were used to create the ‘Fractures’ variable to include all fractures.

### Other measures

Highest level of educational attainment was collected by self-report (primary, secondary, tertiary). The Cut Down, Angry, Guilty, Eye Opener (CAGE) scale was used to assess for excess alcohol intake [25]. Heart disease was defined as self-reported history of angina, heart attack, congestive cardiac failure, murmur and/or arrhythmia. Pain was defined as answering "yes" when asked "Are you often troubled with pain?". History of depression and anxiety were self-reported. Self-reported sleep problems were collected using two items from the Jenkins Sleep Problems Scale [26], "How often do you have trouble falling asleep?" and "How often do you have trouble with waking up too early and not being able to fall asleep again?"; participants were defined as having sleep problems if they responded "most of the time" or "sometimes" to either question. Indications for antipsychotic medication use were defined by self-reported history of hallucinations, schizophrenia, psychosis, mood swings or bipolar disorder. Urinary incontinence was defined by self-report. Heel ultrasound was performed to assess bone density, categorized as normal bone density (T score > -1.0 standard deviations), osteopenia (bone mineral density T score < -2.5 standard deviations), or osteoporosis (bone mineral density T score > -2.5 standard deviations) [27]. Chronic disease burden was assessed by self-report of cancer, liver disease, kidney disease, thyroid disease, arthritis, lung disease, eye conditions and diabetes. Cognitive impairment was defined as self-reporting memory as fair/poor when asked “How would you rate your day-to-day memory at the present time?” and/or Mini Mental State Examination (MMSE) score ≤ 24. Height and weight were measured during health assessments.

### Statistical analysis

Data were analysed using Stata version 15.1 (Stata®, College Station, TX, USA). Baseline characteristics of the study sample by STOPPFall medication use (one or ≥ 2 STOPPFall medications) were presented descriptively using proportions and mean values with 95% confidence intervals (CIs). Continuous variables were compared using Student’s t test and categorical variables were compared using chi-square tests.

Logistic regression models using interaction terms were used to report odds ratios (ORs) with 95% CIs for the association between the interaction of STOPPFall medication use and delayed BP recovery or “any OH” at baseline assessment, and falls (all falls, injurious and unexplained falls) and all fractures at follow-up.

Covariates were chosen a priori to control for medical conditions that STOPPFall medications are prescribed to treat, including heart disease, chronic pain, depression, anxiety, sleep problems, psychosis and related disorders, and urinary incontinence, together with other relevant covariates including age, sex, height, weight, educational attainment, CAGE score (representing alcohol excess), bone mineral density status from heel ultrasound, chronic disease burden, cognitive impairment, previous fracture and follow-up duration.

Separate logistic regression models were fitted for each outcome (all falls, unexplained falls, injurious falls, and fractures). Because these outcomes represent distinct clinical events and the analyses were prespecified, formal adjustments for multiple comparisons were not applied. Results were interpreted primarily based on effect sizes, confidence intervals, and consistency across related outcomes.

### Ethics

The TILDA study was approved by the Faculty of Health Sciences Research Ethics Committee at Trinity College Dublin, and all participants provided informed written consent. All experimental procedures adhered to the Declaration of Helsinki.

## Results

### Baseline characteristics

There were 1390 participants included in this study, mean age at baseline assessment was 71.0 years (95% CI 70.7 – 71.3) and 51% (704/1390) were female. Over one in four participants (372/1390, 26.8%) were prescribed one STOPPFall medication at baseline, with a further 11% (147/1390) prescribed ≥ 2 STOPPFall medications. Delayed BP recovery at 30 s was recorded in 24% (338/1390) of participants at baseline assessment and 32% (446/1390) had “any OH”.

The majority of participants completed follow-up to Wave 3 (1263/1390, 91%), and 9% (127/1390) completed follow-up to Wave 2. Over 40% of participants fell during follow-up (592/1390, 42.6%), 12.1% (168/1390) had an unexplained fall, 21% (288/1390) had an injurious fall, and 12% (160/1390) reported sustaining any fracture during follow-up.

Differences in baseline characteristics between participants by prescription of STOPPFall medication are shown in Table 1. Participants prescribed any STOPPFall medications were significantly older, more likely to report a history of heart disease, chronic pain and depression and less likely to have no chronic diseases, and those prescribed ≥ 2 STOPPFall medications were also significantly more likely to report anxiety, urinary incontinence, sleep problems, an indication for antipsychotic medication use, alcohol excess, higher chronic disease burden, cognitive impairment, and lower level of educational attainment.

**Table 1:** Baseline characteristics (at Wave 1) of total study sample and grouped by STOPPFall medication use

	Total sample (n = 1390)	One STOPPFall medication (n = 372)	≥ 2 STOPPFall medications (n = 147)
Mean age (years) with 95% CI	71.0 (70.7–71.3)	72.0 (71.4–72.5) *	71.8 (71.0–72.6) *
Age bands (proportions with 95% CI)			
65–69 years	0.47 (0.44–0.50)	0.40 (0.35–0.45) *	0.40 (0.32–0.48) *
70–74 years	0.30 (0.28–0.32)	0.30 (0.26–0.35)	0.29 (0.22–0.37)
≥75 years	0.23 (0.21–0.25)	0.30 (0.26–0.35) *	0.31 (0.24–0.39) *
Female sex (proportions with 95% CI)	0.51 (0.48–0.53)	0.51 (0.46–0.56)	0.58 (0.50–0.66)
Educational attainment (proportions with 95% CI)			
Primary	0.30 (0.28–0.33)	0.31 (0.26–0.36)	0.40 (0.32–0.48) * ‡
Secondary	0.37 (0.34–0.39)	0.36 (0.31–0.41)	0.35 (0.28–0.43)
Tertiary	0.33 (0.31–0.36)	0.33 (0.28–0.38)	0.25 (0.18–0.32) *
CAGE score (proportions with 95% CI)			
< 2	0.84 (0.82–0.86)	0.85 (0.81–0.88)	0.77 (0.69–0.83) * ‡
≥ 2	0.09 (0.07–0.10)	0.08 (0.06–0.11)	0.14 (0.09–0.20) *
Did not complete	0.07 (0.06–0.08)	0.07 (0.05–0.10)	0.09 (0.06–0.15)
Heart disease (proportions with 95% CI)	0.26 (0.23–0.28)	0.34 (0.29–0.39) *	0.40 (0.32–0.48) *
Chronic pain (proportions with 95% CI)	0.35 (0.33–0.38)	0.41 (0.36–0.46) *	0.63 (0.54–0.70) * ‡
Depression (proportions with 95% CI)	0.05 (0.03–0.06)	0.07 (0.05–0.10) *	0.17 (0.12–0.24) * ‡
Anxiety (proportions with 95% CI)	0.04 (0.03–0.05)	0.03 (0.02–0.06)	0.14 (0.09–0.21) * ‡
Urinary incontinence (proportions with 95% CI)	0.14 (0.13–0.16)	0.16 (0.13–0.21)	0.28 (0.21–0.36) * ‡
Sleep problems (proportions with 95% CI)	0.57 (0.55–0.60)	0.58 (0.52–0.62)	0.69 (0.61–0.76) * ‡
Indication for antipsychotic medication (proportions with 95% CI)	0.01 (0.005–0.02)	0.01 (0.004–0.03)	0.05 (0.02–0.10) * ‡
Heel ultrasound osteoporosis status, (proportions with 95% CI)			
Normal	0.45 (0.43–0.48)	0.44 (0.39–0.49)	0.40 (0.32–0.48)
Osteopenia	0.41 (0.39–0.44)	0.40 (0.35–0.45)	0.44 (0.36–0.52)
Osteoporosis	0.14 (0.12–0.16)	0.16 (0.13–0.20)	0.16 (0.11–0.23)
Chronic disease number (proportions with 95% CI)			
0	0.39 (0.36–0.41)	0.32 (0.27–0.37) *	0.17 (0.12–0.24) * ‡
1	0.37 (0.35–0.40)	0.42 (0.37–0.47) *	0.43 (0.35–0.51)
≥ 2	0.24 (0.22–0.26)	0.26 (0.22–0.31)	0.40 (0.32–0.48) * ‡
Cognitive impairment (proportions with 95% CI)	0.21 (0.18–0.23)	0.20 (0.17–0.25)	0.29 (0.22–0.37) * ‡
Previous fracture (proportions with 95% CI)	0.15 (0.13–0.17)	0.15 (0.12–0.19)	0.16 (0.11–0.23)
Mean seated systolic blood pressure (mmHg) with 95% CI	139.9 (138.8–141.0)	139.1 (137.1–141.2)	137.4 (134.0–140.8)
Orthostatic hypotension (OH) (proportions with 95% CI)			
Delayed BP Recovery at 30 s	0.24 (0.22–0.27)	0.27 (0.23–0.32)	0.37 (0.29–0.45) * ‡
Any OH (30/60/90/120 s)	0.32 (0.30–0.35)	0.33 (0.28–0.38)	0.43 (0.35–0.51) * ‡

Statistically significant differences between groups using one STOPPFall medication or ≥ 2 STOPPFall medications compared to the total sample (*p* < 0.05) are highlighted with an asterisk (*). Statistically significant differences between groups using one STOPPFall medication compared to ≥ 2 STOPPFall medications (*p* < 0.05) are highlighted as (‡)

Heart disease was defined as a self-reported history of angina, heart attack, congestive cardiac failure, murmur and/or arrhythmia. Chronic pain was defined as answering "yes" when asked "Are you often troubled with pain?". History of depression, anxiety and urinary incontinence were defined by self-report. Sleep problems was defined by positive response to two items from the Jenkins Sleep Problems Scale, "How often do you have trouble falling asleep?" and "How often do you have trouble with waking up too early and not being able to fall asleep again?". Indications for antipsychotic medication use were defined by a self-reported history of hallucinations, schizophrenia, psychosis, mood swings or bipolar disorder. T score from heel ultrasound used to define normal bone density (T score > -1.0 standard deviations), osteopenia (bone mineral density T score < -2.5 standard deviations), or osteoporosis (bone mineral density T score > -2.5 standard deviations). Chronic disease burden was assessed by self-report of cancer, liver disease, kidney disease, thyroid disease, arthritis, lung disease, eye conditions and diabetes. Cognitive impairment was defined as self-reporting memory as fair/poor when asked “How would you rate your day-to-day memory at the present time?” and/or Mini Mental State Examination (MMSE) score ≤ 24. Previous fracture at Wave 1 was defined by self-report. Delayed BP recovery was defined as a drop in systolic BP ≥ 20 mmHg and/or drop in diastolic BP ≥ 10 mmHg at 30 s after standing. Participants were defined as having “any OH” by a drop in systolic BP ≥ 20 mmHg and/or drop in diastolic BP ≥ 10 mmHg at any of 30, 60, 90 or 120 s after standing

STOPPFall medications were identified with the following ATC codes: benzodiazepines (ATC codes N03AE, N05BA and N05CD), antipsychotics (ATC code N05A), benzodiazepine-related drugs (N05CF), opioids (N02A, R05DA), antidepressants (N06A, N06CA), antiepileptics (N03), diuretics (C02L, C03, C07B, C07C, C07D, C08GA, C09BA, C09BX01, C09BX03, C10BX13, C09DA, C09DX01, C09DX03, C09DX06, C09DX07, C09XA52, C09XA54), alpha-blockers used as antihypertensives (C02CA), alpha blockers used for prostate hyperplasia (G04CA), centrally-acting antihypertensives (C02A), antihistamines (N07CA02, R06), vasodilators used in cardiac diseases (C01D), and drugs for urinary frequency and incontinence (G04BD, G04CA53). Anticholinergic medications not already classified in other STOPPFall medication classes included gastrological agents (A03AA07 A03AB05 A03BA01 A03BA03 A03BB01), Parkinsonian agents (N04AA01 N04AA02 N04AA04 N04AB02 N04AC01), antihistamines (N02BE51) and other medications (M03BA03 M03BC01 N05CM05 N05BB01)

### Association of STOPPFall medications and delayed BP recovery with falls and fractures

Falls (including unexplained and injurious falls) and fractures reported among participants in the delayed BP recovery and “any OH” groups are reported in Appendix 1 (Supplementary Data). Table 2 presents the outputs from the fully adjusted logistic regression models showing the association of the interaction of STOPPFall medication use and delayed BP recovery, and STOPPFall medication use and “any OH”, on all falls, unexplained falls, injurious falls and all fractures, compared to participants using no STOPPFall medications and without evidence of these delays in orthostatic BP recovery.

**Table 2:** Fully adjusted logistic regression models reporting odds ratios with 95% confidence intervals with falls and fractures as dependent variables

	All falls		Unexplained falls		Injurious falls		All fractures	
	OR (95% CI)	*p*	OR (95% CI)	*p*	OR (95% CI)	*p*	OR (95% CI)	*p*
STOPPFall Medication x Delayed BP Recovery (ref: 0 STOPPFall medication, Delayed BP recovery -)								
0 STOPPFall medication, Delayed BP recovery +	1.69 (1.20–2.39)	0.003	2.03 (1.25–3.32)	0.005	2.02 (1.35–3.03)	0.001	1.91 (1.17–3.12)	0.010
1 STOPPFall medication, Delayed BP recovery -	1.27 (0.94–1.71)	0.126	1.00 (0.61–1.63)	0.998	1.54 (1.06–2.24)	0.023	1.30 (0.80–2.12)	0.284
1 STOPPFall medication, Delayed BP recovery +	1.47 (0.94–2.31)	0.090	1.37 (0.72–2.60)	0.342	1.13 (0.65–1.98)	0.664	1.41 (0.70–2.82)	0.335
≥ 2 STOPPFall medications, Delayed BP recovery -	1.83 (1.13–2.96)	0.014	1.53 (0.82–2.88)	0.184	1.72 (0.99–2.98)	0.051	1.60 (0.80–3.22)	0.183
≥ 2 STOPPFall medications, Delayed BP recovery +	1.93 (1.04–3.59)	0.036	1.84 (0.86–3.92)	0.115	2.97 (1.55–5.68)	0.001	3.50 (1.66–7.41)	0.001
STOPPFall Medication x "Any OH" (ref: 0 STOPPFall medication, Any OH -)								
0 STOPPFall medication, Any OH +	1.53 (1.12–2.08)	0.007	2.02 (1.28–3.19)	0.003	1.52 (1.04–2.21)	0.030	1.90 (1.20–3.02)	0.006
1 STOPPFall medication, Any OH -	1.30 (0.95–1.78)	0.107	1.10 (0.66–1.84)	0.705	1.47 (0.99–2.17)	0.055	1.37 (0.82–2.29)	0.232
1 STOPPFall medication, Any OH +	1.43 (0.94–2.17)	0.093	1.31 (0.70–2.44)	0.394	1.18 (0.71–1.97)	0.527	1.53 (0.80–2.91)	0.201
≥ 2 STOPPFall medications, Any OH -	1.84 (1.12–3.04)	0.017	1.73 (0.90–3.33)	0.103	1.91 (1.09–3.34)	0.025	2.06 (1.01–4.18)	0.046
≥ 2 STOPPFall medications, Any OH +	1.98 (1.11–3.54)	0.021	1.79 (0.86–3.70)	0.119	2.25 (1.21–4.20)	0.011	2.81 (1.34–5.90)	0.006

STOPPFall Medication x Delayed BP Recovery represents the interaction between STOPPFall medication use and delayed BP recovery (drop in systolic BP ≥ 20 mmHg and/or drop in diastolic BP ≥ 10 mmHg at 30 s after standing) during active stand. Delayed BP recovery + = delayed BP recovery present, Delayed BP recovery–= delayed BP recovery absent

STOPPFall Medication x “Any OH” represents the interaction between STOPPFall medication use and any OH (drop in systolic BP ≥ 20 mmHg and/or drop in diastolic BP ≥ 10 mmHg at any of 30, 60, 90 or 120 s after standing) during active stand. Any OH + = Any OH present, Any OH–= Any OH absent

Fully adjusted logistic regression models reporting odds ratios with 95% confidence intervals with all falls, unexplained falls, injurious falls, and all fractures (comprising hip, wrist, vertebral and other fractures) during follow-up as dependent variables. Models were adjusted for age, sex, height, weight, educational attainment, heart disease, pain, depression, anxiety, sleep problems, indication for antipsychotic medication, urinary incontinence, alcohol excess, osteoporosis status by heel ultrasound, chronic disease burden, cognitive impairment, previous fracture and follow up duration

After controlling for relevant covariates, fully adjusted logistic regression models showed that there was a significant association between delayed BP recovery (without STOPPFall medication use) and all falls (n = 90) [OR 1.69 (95%CI 1.20 – 2.39); *p* = 0.003], use of ≥ 2 STOPPFall medications (without delayed BP recovery) and all falls (n = 54) [OR 1.83 (95%CI 1.13 – 2.96); *p* = 0.014], and use of ≥ 2 STOPPFall medications combined with delayed BP recovery and all falls (n = 33) [OR 1.93 (95%CI 1.04 – 3.59); *p* = 0.036]. The cumulative effect of ≥ 2 STOPPFall medications with delayed BP recovery conferred the highest increased odds of all falls. The full output from the fully adjusted logistic regression model with all falls as the dependent variable is reported in Table 3.

**Table 3:** Fully adjusted logistic regression model with all falls as dependent variable (n = 1390) by interaction of STOPPFall medications and delayed BP recovery

Falls(Any)	OR (95% CI)	Z	P
STOPPFall Medication x Delayed BP Recovery (ref: 0 STOPPFall medications, Delayed BP recovery -)			
0 STOPPFall medication, Delayed BP recovery +	1.69 (1.20–2.39)	2.99	0.003
1 STOPPFall medication, Delayed BP recovery -	1.27 (0.94–1.71)	1.53	0.126
1 STOPPFall medication, Delayed BP recovery +	1.47 (0.94–2.31)	1.69	0.090
≥ 2 STOPPFall medications, Delayed BP recovery -	1.83 (1.13–2.96)	2.46	0.014
≥ 2 STOPPFall medications, Delayed BP recovery +	1.93 (1.04–3.59)	2.10	0.036
Age bands (ref: 65–69 years)			
70–74 years	1.03 (0.79–1.35)	0.25	0.804
≥ 75 years	1.46 (1.09–1.97)	2.50	0.012
Female sex	1.05 (0.74–1.49)	0.25	0.799
Height	0.98 (0.96–1.00)	-1.67	0.094
Weight	0.99 (0.98–1.01)	-0.76	0.449
Educational attainment (ref: Primary)			
Secondary	1.22 (0.92–1.62)	1.39	0.164
Tertiary	1.45 (1.09–1.95)	2.52	0.012
Heart disease	0.87 (0.67–1.14)	-1.02	0.308
Pain	1.46 (1.14–1.87)	2.99	0.003
Depression	1.39 (0.76–2.53)	1.07	0.286
Anxiety	1.28 (0.63–2.61)	0.69	0.492
Sleep problems	1.01 (0.80–1.28)	0.11	0.913
Indication for antipsychotic medication	0.67 (0.20–2.30)	-0.64	0.523
Urinary incontinence	1.31 (0.95–1.81)	1.66	0.098
CAGE Alcohol Scale score (ref: 0–1)			
CAGE ≥ 2	1.36 (0.91–2.03)	1.49	0.136
Did not complete	1.04 (0.67–1.62)	0.19	0.847
Heel ultrasound osteoporosis status (ref: normal)			
Osteopenia	1.06 (0.82–1.37)	0.46	0.646
Osteoporosis	1.01 (0.69–1.47)	0.04	0.966
Chronic disease number (ref: 0)			
1	1.20 (0.92–1.56)	1.34	0.182
≥ 2	1.66 (1.22–2.26)	3.20	0.001
Cognitive impairment	1.21 (0.91–1.60)	1.32	0.188
Previous fracture	1.21 (0.88–1.65)	1.17	0.241
Follow up duration (ref: Wave 2)			
Wave 3	1.46 (0.98–2.18)	1.85	0.064

STOPPFall Medication x Delayed BP Recovery represents the interaction between STOPPFall medication use and delayed BP recovery (drop in systolic BP ≥ 20 mmHg and/or drop in diastolic BP ≥ 10 mmHg at 30 s after standing) during active stand. Delayed BP recovery + = delayed BP recovery present, Delayed BP recovery–= delayed BP recovery absent

Heart disease was defined as a self-reported history of angina, heart attack, congestive cardiac failure, murmur and/or arrhythmia. Chronic pain was defined as answering "yes" when asked "Are you often troubled with pain?". History of depression, anxiety and urinary incontinence were defined by self-report. Sleep problems was defined by positive response to two items from the Jenkins Sleep Problems Scale, "How often do you have trouble falling asleep?" and "How often do you have trouble with waking up too early and not being able to fall asleep again?". Indications for antipsychotic medication use were defined by a self-reported history of hallucinations, schizophrenia, psychosis, mood swings or bipolar disorder. T score from heel ultrasound used to define normal bone density (T score > -1.0 standard deviations), osteopenia (bone mineral density T score < -2.5 standard deviations), or osteoporosis (bone mineral density T score > -2.5 standard deviations). Chronic disease burden was assessed by self-report of cancer, liver disease, kidney disease, thyroid disease, arthritis, lung disease, eye conditions and diabetes. Cognitive impairment was defined as self-reporting memory as fair/poor when asked “How would you rate your day-to-day memory at the present time?” and/or Mini Mental State Examination (MMSE) score ≤ 24. Previous fracture at Wave 1 was defined by self-report

There was a significant association between delayed BP recovery (without STOPPFall medication use) and unexplained falls (n = 30) [OR 2.03 (95%CI 1.25 – 3.32); *p* = 0.005]. No other combinations of STOPPFall medication use and delayed BP recovery reached statistical significance for unexplained falls.

There was a significant association between delayed BP recovery (without STOPPFall medication use) and injurious falls (n = 52) [OR 2.02 (95%CI 1.35 – 3.03); *p* = 0.001], use of one STOPPFall medication (without delayed BP recovery) and injurious falls (n = 62) [OR 1.54 (95%CI 1.06 – 2.24); *p* = 0.023], with the combination of ≥ 2 STOPPFall medications with delayed BP recovery conferring the highest odds of injurious falls (n = 22) [OR 2.97 (95%CI 1.55 – 5.68); *p* = 0.001].

**Fig. 1:**
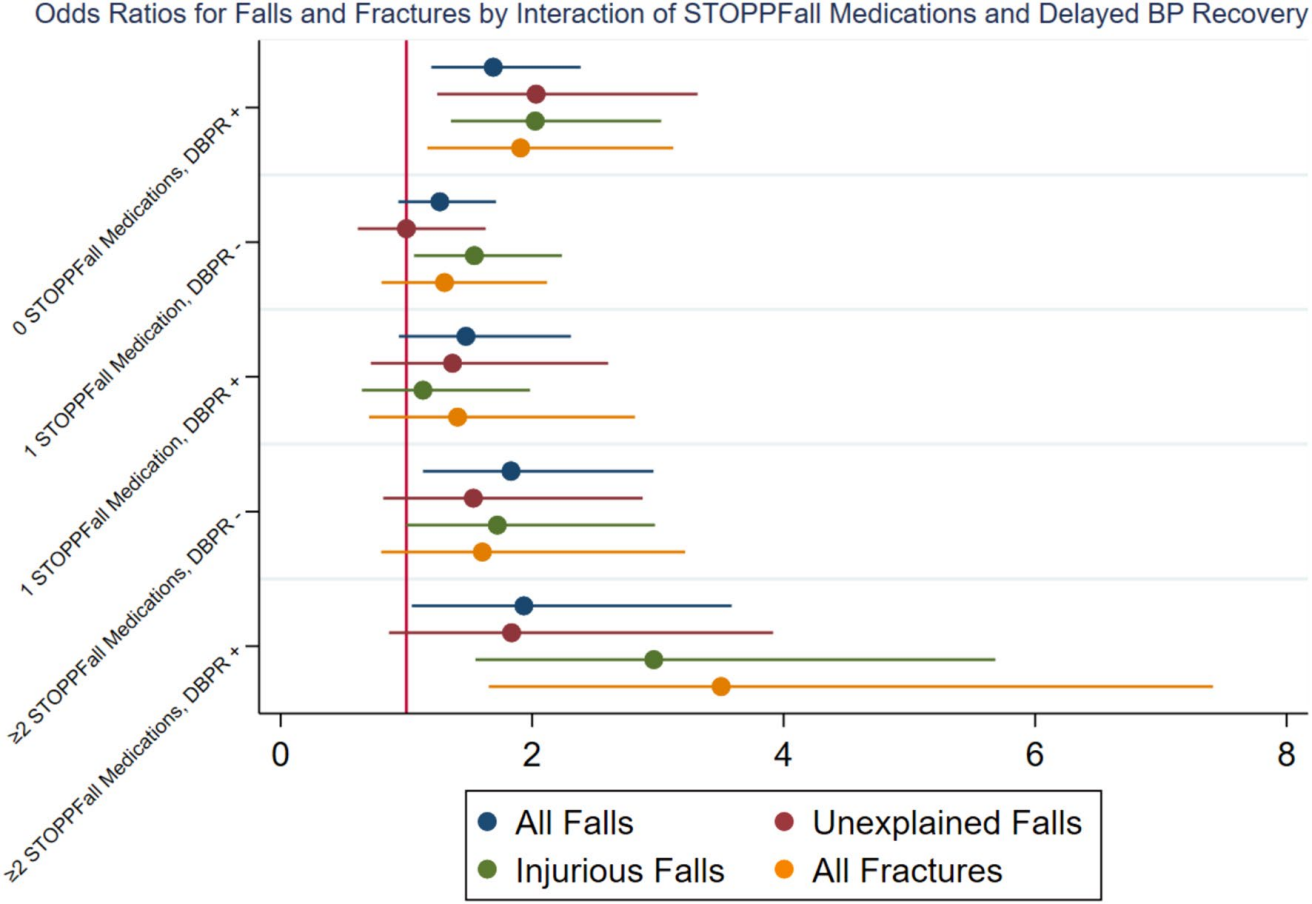
Odds ratios (with 95% confidence intervals) for falls and fractures by interaction of STOPPFall medications and delayed BP recovery. Data presented are odds ratios with 95% confidence intervals from fully adjusted logistic regression models with all falls, unexplained falls, injurious falls and all fractures as dependent variables. Models were adjusted for age, sex, height, weight, educational attainment, heart disease, pain, depression, anxiety, sleep problems, indication for antipsychotic medication, urinary incontinence, alcohol excess, osteoporosis status by heel ultrasound, chronic disease burden, cognitive impairment, previous fracture and follow up duration. Delayed BP recovery was defined as a drop in systolic BP ≥ 20 mmHg and/or drop in diastolic BP ≥ 10 mmHg at 30 s after standing. *DBPR* delayed BP recovery, DBPR + = delayed BP recovery present, DBPR–= delayed BP recovery absent

The presence of delayed BP recovery (without STOPPFall medication use) significantly predicted all fractures (n = 30) [OR 1.91 (95%CI 1.17 – 3.12);*p* = 0.010], and the combination of ≥ 2 STOPPFall medications with delayed BP recovery demonstrated a higher odds of all fractures (n = 14) [OR 3.50 (95%CI 1.66 – 7.41);*p* = 0.001]. Odds ratios with 95% CIs for all falls, unexplained falls, injurious falls, and all fractures, by interaction of STOPPFall medications and delayed BP recovery, is shown in Fig. 1.

### Association of STOPPFall medications and “any OH” with falls and fractures

After controlling for relevant covariates, fully adjusted logistic regression models showed that there was a significant association between any OH (without STOPPFall medication use) and all falls (n = 120) [OR 1.53 (95%CI 1.12 – 2.08); *p* = 0.007], use of ≥ 2 STOPPFall medications (without any OH) and all falls (n = 48) [OR 1.84 (95%CI 1.12 – 3.04); *p* = 0.017], and the cumulative effect of ≥ 2 STOPPFall medications combined with any OH conferring the highest increased odds of all falls (n = 39) [OR 1.98 (95%CI 1.11 – 3.54); *p* = 0.021]. The full output from the fully adjusted logistic regression model with all falls as the dependent variable is reported in Table 4.

**Table 4:** Fully adjusted logistic regression model with all falls as dependent variable (n = 1390) by interaction of STOPPFall medications and “Any OH”

Falls(Any)	OR (95% CI)	Z	P
STOPPFall Medication x "Any OH" (ref: 0 STOPPFall medications, Any OH -)			
0 STOPPFall medication, Any OH +	1.53 (1.12–2.08)	2.69	0.007
1 STOPPFall medication, Any OH -	1.30 (0.95–1.78)	1.61	0.107
1 STOPPFall medication, Any OH +	1.43 (0.94–2.17)	1.68	0.093
≥ 2 STOPPFall medications, Any OH -	1.84 (1.12–3.04)	2.39	0.017
≥ 2 STOPPFall medications, Any OH +	1.98 (1.11–3.54)	2.31	0.021
Age bands (ref: 65–69 years)			
70–74 years	1.04 (0.80–1.35)	0.28	0.783
≥ 75 years	1.48 (1.10–1.99)	2.59	0.010
Female sex	1.05 (0.74–1.50)	0.29	0.770
Height	0.98 (0.96–1.00)	-1.65	0.099
Weight	0.99 (0.98–1.01)	-0.74	0.460
Educational attainment (ref: Primary)			
Secondary	1.22 (0.92–1.62)	1.40	0.163
Tertiary	1.45 (1.09–1.95)	2.52	0.012
Heart disease	0.87 (0.66–1.13)	-1.06	0.291
Pain	1.47 (1.14–1.88)	3.03	0.002
Depression	1.39 (0.76–2.53)	1.07	0.285
Anxiety	1.27 (0.63–2.59)	0.67	0.503
Sleep problems	1.01 (0.80–1.28)	0.10	0.921
Indication for antipsychotic medication	0.68 (0.20–2.33)	-0.61	0.540
Urinary incontinence	1.30 (0.94–1.80)	1.60	0.110
CAGE Alcohol Scale score (ref: 0–1)			
CAGE ≥ 2	1.35 (0.91–2.02)	1.48	0.139
Did not complete	1.04 (0.67–1.61)	0.16	0.869
Heel ultrasound osteoporosis status (ref: normal)			
Osteopenia	1.05 (0.81–1.36)	0.38	0.702
Osteoporosis	1.02 (0.70–1.49)	0.11	0.916
Chronic disease number (ref: 0)			
1	1.18 (0.90–1.54)	1.22	0.224
≥ 2	1.65 (1.21–2.24)	3.17	0.002
Cognitive impairment	1.21 (0.91–1.60)	1.31	0.191
Previous fracture	1.21 (0.88–1.65)	1.16	0.245
Follow up duration (ref: Wave 2)			
Wave 3	1.44 (0.96–2.14)	1.78	0.076

STOPPFall Medication x “Any OH” represents the interaction between STOPPFall medication use and any OH (drop in systolic BP ≥ 20 mmHg and/or drop in diastolic BP ≥ 10 mmHg at any of 30, 60, 90 or 120 s after standing) during active stand. Any OH + = Any OH present, Any OH–= Any OH absent

Heart disease was defined as a self-reported history of angina, heart attack, congestive cardiac failure, murmur and/or arrhythmia. Chronic pain was defined as answering "yes" when asked "Are you often troubled with pain?". History of depression, anxiety and urinary incontinence were defined by self-report. Sleep problems was defined by positive response to two items from the Jenkins Sleep Problems Scale, "How often do you have trouble falling asleep?" and "How often do you have trouble with waking up too early and not being able to fall asleep again?". Indications for antipsychotic medication use were defined by a self-reported history of hallucinations, schizophrenia, psychosis, mood swings or bipolar disorder. T score from heel ultrasound used to define normal bone density (T score > -1.0 standard deviations), osteopenia (bone mineral density T score < -2.5 standard deviations), or osteoporosis (bone mineral density T score > -2.5 standard deviations). Chronic disease burden was assessed by self-report of cancer, liver disease, kidney disease, thyroid disease, arthritis, lung disease, eye conditions and diabetes. Cognitive impairment was defined as self-reporting memory as fair/poor when asked “How would you rate your day-to-day memory at the present time?” and/or Mini Mental State Examination (MMSE) score ≤ 24. Previous fracture at Wave 1 was defined by self-report

There was a significant association between any OH (without STOPPFall medication use) and unexplained falls (n = 40) [OR 2.02 (95%CI 1.28 – 3.19); *p* = 0.003]. No other combinations of STOPPFall medication use and any OH reached statistical significance for unexplained falls.

There was a significant association between any OH (without STOPPFall medication use) and injurious falls (n = 62) [OR 1.52 (95%CI 1.04 – 2.21);*p* = 0.030], use of ≥ 2 STOPPFall medications (without any OH) and injurious falls (n = 26) [OR 1.91 (95%CI 1.09 – 3.34);*p* = 0.025], and the combination of ≥ 2 STOPPFall medications with any OH conferring the highest odds of injurious falls (n = 23) [OR 2.25 (95%CI 1.21 – 4.20); *p* = 0.011].

**Fig. 2:**
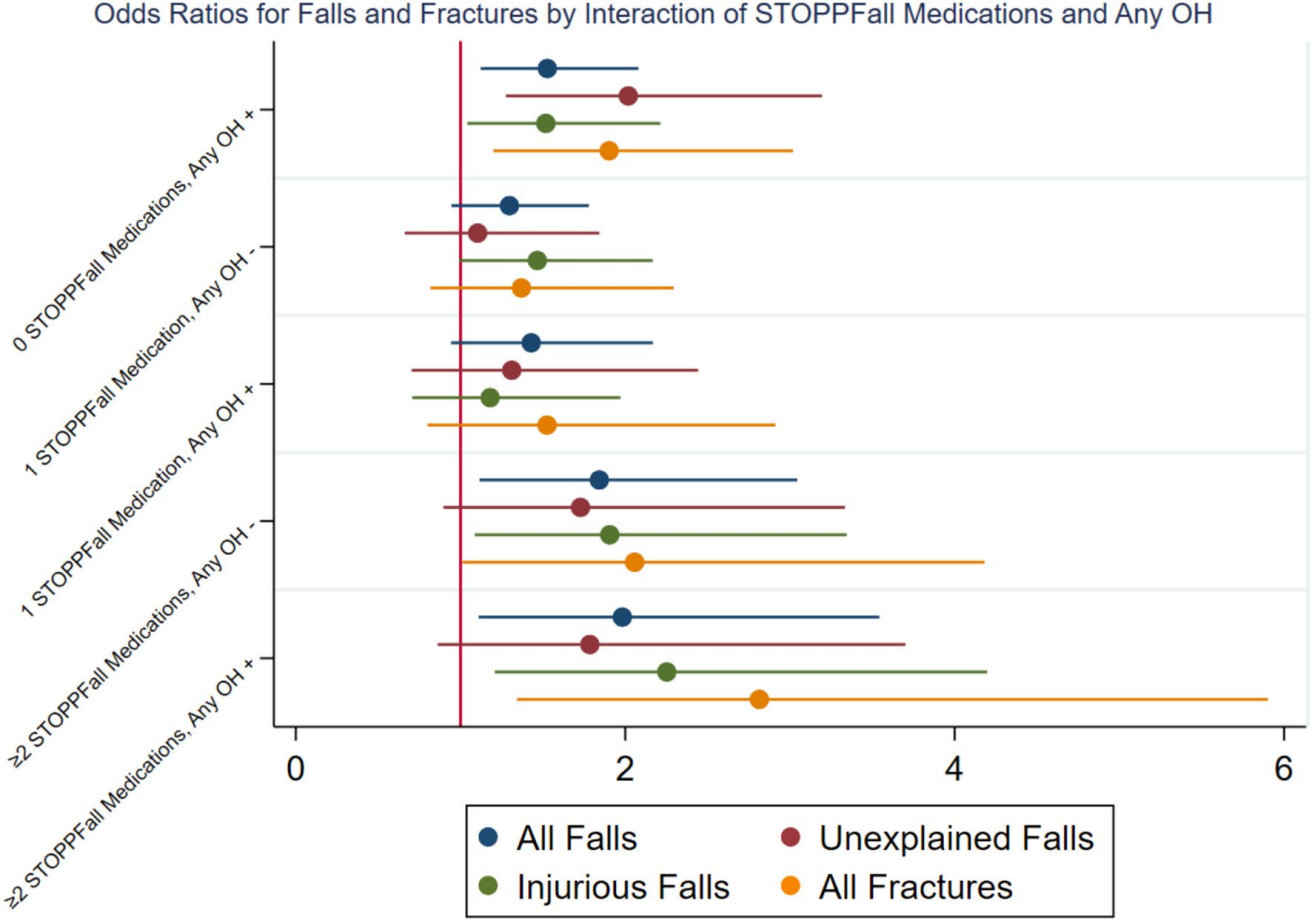
Odds ratios (with 95% confidence intervals) for falls and fractures by interaction of STOPPFall medications and any OH. Data presented are odds ratios with 95% confidence intervals from fully adjusted logistic regression models with all falls, unexplained falls, injurious falls and all fractures as dependent variables. Models were adjusted for age, sex, height, weight, educational attainment, heart disease, pain, depression, anxiety, sleep problems, indication for antipsychotic medication, urinary incontinence, alcohol excess, osteoporosis status by heel ultrasound, chronic disease burden, cognitive impairment, previous fracture and follow up duration. Participants were defined as having “any OH” by a drop in systolic BP ≥ 20 mmHg and/or drop in diastolic BP ≥ 10 mmHg at any of 30, 60, 90 or 120 s after standing. *OH* orthostatic hypotension, Any OH + = Any OH present, Any OH–= Any OH absent

There was a significant association between any OH (without STOPPFall medication use) and all fractures (n = 40) [OR 1.90 (95%CI 1.20 – 3.02);*p* = 0.006], use of ≥ 2 STOPPFall medications (without any OH) and all fractures (n = 14) [OR 2.06 (95%CI 1.01 – 4.18);*p* = 0.046], with the combination of ≥ 2 STOPPFall medications and any OH demonstrating the highest odds of all fractures (n = 14) [OR 2.81 (95%CI 1.34 – 5.90); *p* = 0.006]. Odds ratios with 95% CIs for all falls, unexplained falls, injurious falls, and all fractures, by interaction of STOPPFall medications and any OH, is shown in Fig. 2.

## Discussion

This study demonstrates that the combination of ≥ 2 STOPPFall medications and delayed BP recovery is independently associated with all falls, injurious falls and all fractures in a large cohort of community dwelling older people, conferring increased odds of these outcomes compared to the presence of delayed BP recovery in isolation. Prescription of ≥ 2 STOPPFall medications combined with delayed BP recovery was associated with more than three-fold higher odds of all fractures during follow-up, compared with participants using no STOPPFall medications without evidence of delayed BP recovery.

Presence of any OH, without STOPPFall medication use, is independently associated with all falls, unexplained falls, injurious falls and all fractures, and prescription of ≥ 2 STOPPFall medications without any OH is independently associated with all falls, injurious falls and all fractures; the cumulative effect of prescription of ≥ 2 STOPPFall medications combined with any OH conferred the highest odds of all falls, injurious falls and all fractures, with a greater than two-fold higher odds of both injurious falls and all fractures during follow-up in this cohort, compared with participants using no STOPPFall medications without evidence of any OH.

Individual medication classes within STOPPFall may increase falls risk by causing OH [28], though other side effects caused by these medications may also increase falls risk, such as opioids and benzodiazepines causing sedation [29, 30], antidepressants causing impaired balance [31], or drug-induced movement disorders caused by antipsychotic medication [32]. STOPPFall medication use is common among people who have fallen, with a recent report from TILDA outlining that nearly half of participants who had a fall requiring medical attention were prescribed at least one STOPPFall medication, with over 20% using more than one STOPPFall medication [33]. A recent systematic review and meta-analysis also demonstrated that, among people who have fallen, the pooled prevalence of potentially inappropriate medications and FRIDs at the time of the fall was 68.6% [34].

Evidence of the interaction between STOPPFall medications and OH has so far been limited. A previous study from the Netherlands demonstrated that OH significantly improved on tilt-table testing after withdrawing FRIDs [35], though the greatest effect was among those where cardiovascular FRIDs were withdrawn, and relatively few antihypertensives are included in STOPPFall. Studies assessing STOPPFall medication have so far outlined their association with increased risk of falls [36, 37], with our own studies from TILDA outlining their association with falls and fractures [13] and significantly delayed BP recovery on standing and OH [14]. This is the first study to assess the interaction of STOPPFall medication use with OH and future falls and fractures in older people.

These findings are important because they underscore the importance of multifactorial assessment for falls. Though OH and STOPPFall medication use are important independent risk factors, it is clear that there is amplification of falls risk when they co-exist so should not be considered in isolation. There are of course many other factors beyond STOPPFall medications or OH that can contribute to falls that should be assessed as part of a multifactorial falls risk assessment, such as cognitive impairment, gait and balance disorders, vision problems and environmental risk factors [6].

Consideration should be given to balancing an appropriate indication for prescription of STOPPFall medications with their potential adverse effects, including falls and fractures. Though psychotropic medications, for example, can lead to OH and sedation which can frequently lead to falls in older people [31], they can be necessary in managing symptoms of depression, anxiety and psychosis [38–40] among older people. It is therefore important to regularly re-assess the risk–benefit ratios of all STOPPFall medications to ensure there is an ongoing need for their prescription. Though this can be challenging, STOPPFall was used to develop an online deprescribing tool, a useful resource for clinicians providing practical advice for medication withdrawal strategies, when this is appropriate [12].

It is also important to consider that there may be multiple contributing factors for OH among older people beyond STOPPFall medication use. It is notable that this study demonstrated that both delayed BP recovery and any OH, without STOPPFall medication use, was independently associated with all falls, unexplained falls, injurious falls and all fractures. Though medication use is the most common cause of non-neurogenic OH [41], this can also be caused by other factors such as volume depletion and heart failure, and neurogenic OH can be caused by primary (e.g. idiopathic Parkinson's disease) or secondary (e.g. diabetes mellitus) autonomic dysfunction. These factors should also be addressed and optimised alongside deprescribing STOPPFall medications when OH is detected, and education and lifestyle advice including adequate hydration and salt intake remain the cornerstone of first-line treatment for OH.

There are some limitations to this study. Data relating to falls and fractures, and medical histories of heart disease, depression, anxiety, sleep problems and chronic diseases are based on self-report, and could be subject to recall bias. Data relating to medication is limited by unavailability of medication dosage and inability to confirm compliance with medications. There were insufficient numbers of participants using each individual class of STOPPFall medications to consider their effects in isolation, or to assess how different medication classes in combination may impact the cumulative risk of falls or fractures with concurrent OH. Our study included participants with longitudinal follow-up only, raising the possibility of selection/survivor bias, but analysis of baseline characteristics of participants who completed Wave 1 only showed no significant difference in mean age, educational attainment, proportion of participants using STOPPFall medications, delayed BP recovery, any OH or chronic disease burden. Due to the longitudinal nature of the study, information regarding dynamic medication changes between waves of data collection is lacking, which may influence the interpretation of longitudinal associations between medication changes, OH risk, and adverse outcomes including falls and fractures. Some estimates, particularly for fractures and injurious falls, were associated with relatively wide confidence intervals. This likely reflects the relatively small number of events within some exposure strata after stratification by orthostatic hypotension and STOPPFall medication exposure, which reduces statistical precision. Heel ultrasonography was used to assess bone density as it was not feasible to perform dual-energy X-ray absorptiometry (DEXA), the gold standard for bone density assessment, on all participants. Strengths include the large study sample, use of Finometer® Midi for detailed continuous orthostatic BP measurement during active stand, and robust adjustment for relevant covariates that may contribute to OH, falls and fractures, including controlling for indications for STOPPFall medication use.

In conclusion, this study shows that prescription of ≥ 2 STOPPFall medications combined with OH, is independently associated with future falls, and fractures, in a large sample of community dwelling older people. The interaction of these factors conferred an increased odds of future falls and fractures compared to their odds of these outcomes in isolation. This highlights the importance of a comprehensive multifactorial falls assessment with multidomain interventions among older people at risk of falls to reduce future burden of falls and fractures, and the associated negative outcomes associated with these events.

## Supplementary Information

Below is the link to the electronic supplementary material.

Supplementary file1 (DOCX 17 KB)

## Data Availability

TILDA offers access to the datasets for research use through pseudonymised publicly accessible dataset files, and through an on-site and remotely accessible Hot Desk Facility. Researchers interested in using regular waves of TILDA data may access the data for free from the following site: Irish Social Science Data Archive (ISSDA) at University College Dublin (http://www.ucd.ie/issda/data/tilda/). Replication of the results reported in this article requires access to the full TILDA dataset. Researchers seeking access to the full TILDA dataset may apply to access the data (tilda.tcd.ie).
