# Supplementary material for: The combined association of STOPPFall medication use and orthostatic blood pressure abnormalities with future falls and fractures in community-dwelling older people
Source: Eur Geriatr Med. 2026 Apr 13;17(3):1229–41. doi: 10.1007/s41999-026-01473-3 (PMC13309396; doi:10.1007/s41999-026-01473-3)
Supplement: Supplementary file 1 — Supplementary file1 (DOCX 17 KB) [file 41999_2026_1473_MOESM1_ESM.docx]

**The combined association of STOPPFall medication use and orthostatic blood pressure abnormalities with future falls and fractures in community-dwelling older people**

**Appendix 1**

Falls (including unexplained and injurious falls) and fractures reported among participants in the delayed BP recovery and “any OH” groups

|  | All Falls (n, %) | Unexplained Falls (n, %) | Injurious Falls (n, %) | All Fractures (n, %) |
| --- | --- | --- | --- | --- |
| Delayed BP Recovery  n = 338 | 173 (51.2) | 58 (17.2) | 94 (27.8) | 56 (16.6) |
| 0 STOPPFall medication, Delayed BP recovery +  n = 184 | 90 (48.9) | 30 (16.3) | 52 (28.3) | 30 (16.3) |
| 1 STOPPFall medication, Delayed BP recovery -  n = 272 | 119 (43.8) | 30 (11.0) | 62 (22.8) | 30 (11.0) |
| 1 STOPPFall medication, Delayed BP recovery +  n = 100 | 50 (50) | 15 (15) | 20 (20) | 12 (12) |
| ≥ 2 STOPPFall medications, Delayed BP recovery -  n = 93 | 54 (58.1) | 20 (21.5) | 27 (29.0) | 14 (15.1) |
| ≥ 2 STOPPFall medications, Delayed BP recovery +  n = 54 | 33 (61.1) | 13 (24.1) | 22 (40.7) | 14 (25.9) |
| Any OH  n = 446 | 219 (49.1) | 72 (16.1) | 111 (24.9) | 69 (15.5) |
| 0 STOPPFall medication, Any OH +  n = 260 | 120 (46.2) | 40 (15.4) | 62 (23.9) | 40 (15.4) |
| 1 STOPPFall medication, Any OH -  n = 249 | 109 (43.8) | 28 (11.2) | 56 (22.5) | 27 (10.8) |
| 1 STOPPFall medication, Any OH +  n = 123 | 60 (48.8) | 17 (13.8) | 26 (21.1) | 15 (12.2) |
| ≥ 2 STOPPFall medications, Any OH -  n = 84 | 48 (57.1) | 18 (21.4) | 26 (31.0) | 14 (16.7) |
| ≥ 2 STOPPFall medications, Any OH +  n = 63 | 39 (61.9) | 15 (23.8) | 23 (36.5) | 14 (22.2) |

Notes:

Delayed BP Recovery defined by drop in systolic BP ≥20 mmHg and/or drop in diastolic BP ≥10 mmHg at 30 seconds after standing during active stand. Delayed BP recovery + = delayed BP recovery present, Delayed BP recovery - = delayed BP recovery absent.

“Any OH” defined by drop in systolic BP ≥20 mmHg and/or drop in diastolic BP ≥10 mmHg at any of 30, 60, 90 or 120 seconds after standing during active stand. Any OH + = Any OH present, Any OH - = Any OH absent.
